# Chinese integrated guideline on the management of gastric precancerous conditions and lesions

**DOI:** 10.1186/s13020-022-00677-6

**Published:** 2022-12-14

**Authors:** Ping Wang, Peng Li, Yingxuan Chen, Li Li, Yuanyuan Lu, Weixun Zhou, Liqun Bian, Beihua Zhang, Xiaolan Yin, Junxiang Li, Jie Chen, Shutian Zhang, Yongquan Shi, Xudong Tang

**Affiliations:** 1 China Academy of Chinese Medical Sciences, Xiyuan Hospital, Beijing, China; 2 Capital Medical University Affiliated Beijing Friendship Hospital, Beijing, China; 3 Shanghai Jiao Tong University School of Medicine Affiliated Renji Hospital, Shanghai, China; 4 China Academy of Chinese Medical Sciences, Guanganmen Hospital, Beijing, China; 5 Air Force Medical University Xijing Hospital, Xi’an, China; 6 Peking Union Medical College Hospital, Beijing, China; 7 Beijing University of Chinese Medicine School of Traditional Chinese Medicine, Beijing, China

**Keywords:** Gastric precancerous conditions, Gastric precancerous lesions; integrated medicine; diagnosis, Treatment, Surveillance

## Abstract

The standardized diagnosis and management of gastric precancerous conditions and lesions are important to prevent gastric cancer. This guideline, created by 5 traditional Chinese medicine and Western medicine associations, based on the current morbidity and diagnosis and treatment of gastric precancerous conditions and lesions, provides specific key points and strategies for diagnosis and treatment in the following five aspects: definition and epidemiology, diagnosis and stage, surveillance, treatment and efficacy evaluation. It is hoped that these aspects, assessed by integrating Western medicine and traditional Chinese medicine and involving multidisciplinary participation, will play a guiding role in clinical diagnosis and treatment and achieve effective secondary prevention of gastric cancer.

## Background

China has a high incidence of gastric cancer [1, 2], with approximately 679,000 new cases of gastric cancer and 498,000 deaths occurring every year. The morbidity and mortality of gastric cancer increase with age [3], posing a serious threat to people's health and causing a huge medical burden. Early detection and treatment of precancerous lesions of gastric cancer, including precancerous diseases (precancerous conditions) and precancerous lesions of gastric cancer (PLGC), is an effective measure to prevent the development of gastric cancer.

The Correa model of gastric adenocarcinoma is generally recognized by the academic community [4]. Much work has been done, and certain achievements and consensus have been achieved in the fields of traditional Chinese medicine and Western medicine on the diagnosis and treatment of *Helicobacter pylori* (*H. pylori*), gastric cancer risk stratification based on atrophy of the gastric mucosa and IM, and treatment and evaluation of PLGC. It is necessary to organize the knowledge about precancerous lesions of gastric cancer and make reasonable primary and secondary prevention programs. The successful publishing of the Management of epithelial precancerous conditions and lesions in the stomach (MAPS II) [5] guidelines,updated in 2019, and the Chinese consensus on the management of gastric epithelial precancerous conditions and lesions (2020) [6] reflect the great importance of the standardized diagnosis and treatment of gastric precancerous lesions in the field of gastroenterology. Previously, relevant contents were reported in the Chinese Consensus on Chronic Gastritis (2017) [7], Expert Consensus on the standardized diagnosis and treatment of gastric low-grade intraepithelial neoplasia (2019) [8], Consensus on the clinical application of gastric mucosal targeted biopsy technology (2018) [9],and Consensus opinions of Traditional Chinese Medicine (TCM) experts in the diagnosis and treatment of chronic gastritis (2017) [10]. This guideline was established by organizing and integrating the data on precancerous lesions in the fields of traditional Chinese medicine and Western medicine from the Digestive Tumor Cooperative Group of Spleen and Stomach Diseases Branch of the China Association of Traditional Chinese Medicine and the Gastroenterology Branch of Chinese Medical Association as the co-lead agency and provided as a reference for clinical practice.

The literature search and evaluation were carried out according to the topics determined by the guideline working group based on discussion. The exposure draft was written based on the extracted data after compositing, discussing and modifying the data. The quality of clinical evidence was assessed as high, middle and low using the Grading of Recommendations Assessment, Development and Evaluation (GRADE) system. The recommendation level was determined through discussion by experts. Then, the preliminary draft was revised many times according to the responses and opinions obtained from two rounds of Delphi expert consultation. The final expert consensus meeting was held on June 5, 2020. These guidelines were established by the votes of 58 gastroenterologists from all over the country, which were based on the full discussion of each statement reported by the experts in the writing group. The statement for which agreement was obtained from all or more than 80% of the members could be adopted; otherwise, a second discussion and vote was conducted by all members, and a third vote decided whether the statement for which the above-mentioned standard was accepted or rejected. This guideline included a total of 48 recommendations involved in five sections: definition and epidemiology, diagnosis and stage, monitoring, treatment, and efficacy evaluation (Table 1).

**Table 1:** Recommendations for the guideline

Section	Item	Statement	Evidence level	Recommendation	Agreement
Definition and epidemiology	Epidemiology	S1. The prevalence rate of chronic atrophic gastritis in China is relatively high and increases with age	Moderate	Strong	100%
		S2. The occurrence of chronic atrophic gastritis is closely related to *H. pylori* infection	High	Strong	100%
		S3. The sensitivity of white light endoscopy in diagnosing atrophic gastritis is poor, and the coincidence rate with the pathological diagnosis is low	Moderate	Weak	96.4%
	Syndrome distribution of gastric precancerous lesions	S4. There is a lack of consolidated standards of gastric precancerous lesions for syndrome differentiation	Low	Weak	92.7%
Diagnosis and grading	Endoscopy diagnosis	S5. The application of defoaming agents and mucolytic agents can improve the visibility of gastric mucosa, which is helpful for detecting gastric mucosal lesions	High	Strong	100%
		S6. When gastric peristalsis severely affects the observation, proper antispasmodic treatment can improve the visual field of observation and facilitate the detection of lesions	Low	Strong	100%
		S7. Intraoperative sedatives are recommended for patients with severe anxiety	Low	Strong	98.2%
		S8. Conventional white-light endoscopy could be used for PLGC screening. High-definition conventional chromoendoscopy, virtual chromoendoscopy and magnifying endoscopy should be used for the diagnosis of patients who are at high risk for gastric carcinoma	High	Strong	100%
		S9. The evaluation and diagnosis of *H. pylori* infection status should be included in endoscopic examinations	Moderate	Strong	100%
	Biopsy	S10. An adequate number, depth and size of biopsy samples are essential for the accurate diagnosis and evaluation of PLGC	Low	Strong	100%
		S11. Targeting biopsy of suspicious lesions is conducive to the evaluation of curative effects and follow-up monitoring	Low	Strong	100%
		S12. Additional biopsies are required for visible lesions and suspected neoplastic lesions under endoscopy	Low	Strong	100%
	Pathological diagnosis and evaluation	S13. Standardizing the protocol of biopsy specimen processing will help improve the accuracy of pathological diagnosis	Low	Strong	100%
		S14.Subtyping incomplete intestinal metaplasia has clinical significance	Low	Strong	98.2%
		S15. Gastric dysplasia needs to be differentiated from reactive hyperplasia	Low	Strong	98.2%
	Mucosa Syndrome Differentiation under Gastroscopy	S16. Mucosal syndrome differentiation under gastroscopy is an extension of inspection in traditional Chinese medicine. It focuses on discriminating gastric mucosal lesions. It is an important reference for the overall syndrome differentiation of traditional Chinese medicine and an objective basis for guiding local treatment	Low	Weak	92.7%
Monitoring	Dysplasia	S17. Patients with dysplasia found on random biopsy should be re-evaluated by high-definition virtual or dyeing chromoendoscopy. If no visible lesions are found in the re-evaluation, the patient should be monitored once again by high-definition virtual or dyeing chromoendoscopy, with an interval of 6–12 months	Low	Strong	100%
		S18. Screening and monitoring of dysplasia under endoscopy should be given great attention	Moderate	Strong	100%
		S19. Patients with uncertain dysplasia diagnosed by non-targeted biopsy can benefit from reevaluation with endoscopic examination	Moderate	Strong	100%
		S20. In patients with high-grade dysplasia, immediate high-quality endoscopic reassessment with Chromoendoscopy (virtual or dye-based) is recommended to determine whether endoscopic or surgical treatment should be performed	Moderate	Strong	98.18%
	CAG and IM	S21. Patients with high-risk atrophic gastritis should be followed up with high-quality endoscopy or white-light endoscopy combined with biopsy every year, especially those with a family history of gastric cancer, who need more intensive follow-up	Low	Weak	100%
		S22. Patients with low-risk atrophic gastritis should be followed up with endoscopy every 3 years, and those with a family history of gastric cancer should be followed up every 1–2 years	Low	Weak	98.2%
	Autoimmune gastritis	S23. Patients with autoimmune gastritis may benefit from endoscopic follow-up every 3 years	Low	Weak	96.36%
	Noninvasive screening method	S24. PGI, PGI/II (PGR), G-17 and *H. pylori* -IgG can be used to screen CAG patients at high-risk of gastric cancer from general population	High	Strong	100%
		S25. Histological and serological MG7 testing can be used to assist in the screening of groups at high-risk of gastric cancer	Moderate	Strong	90.91%
	Risk monitoring of precancerous lesions of gastric cancer by combining disease and syndrome	S26. In carrying out risk monitoring and management with integrated traditional Chinese and Western medicine, in addition to serology, the Kimura-Takemoto classification, OLGA/OLGIM risk assessment, and TCM syndromes can be included	Low	Weak	94.55%
Treatment	Orientation of intervention	S27. Atrophy, intestinal metaplasia and obvious active inflammation can be treated by eradicating *H. pylori* (if positive) and the short-term use of proton pump inhibitors (PPIs) or gastro-protecting agents	Low	Strong	100%
		S28. Chronic atrophic gastritis of the operative link on gastric atrophy (OLGA) and operative link on gastric intestinal metaplasia (OLGIM) stages III/IV are targets of internal medicine interventions	Moderate	Strong	98.2%
		S29. Medical intervention is required for low-grade dysplasia, and endoscopy treatment is required for high-grade dysplasia and some low-grade dysplasia with visible lesions	Moderate	Strong	98.18%
		S30. Surveillance and interventions should be included for indefinite for neoplasm/dysplasia lesions, and pathological consultation can be conducted. A biopsy can be repeated, if necessary, to confirm the diagnosis	Moderate	Strong	98.2%
		S31. Patients with HGD or early gastric cancer can be treated with a combination of Chinese and Western medicine after endoscopic treatment	Moderate	Strong	100%
	Eliminating the risk factors	S32. There is no definite evidence that PPIs can induce or aggravate gastric precancerous lesions such as atrophic gastritis or intestinal metaplasia, but the long-term use of PPI preparations is not recommended in clinical practice	Low	Strong	100%
		S33. A high-salt diet is a risk factor for gastric precancerous lesions. Patients with gastric precancerous lesions should avoid high-salt and pickled foods	Moderate	Strong	100%
		S34. A history of long-term smoking significantly increases the risk of the occurrence and progression of gastric precancerous lesions. Patients with gastric precancerous lesions should quit smoking	High	Strong	100%
		S35. Bile reflux is a risk factor for intestinal metaplasia, and interventions targeting bile reflux may be beneficial to block the occurrence and progression of gastric precancerous lesions	High	Strong	98.2%
	Eradication of *H. pylori*	S36. The eradication of *H. pylori* can prevent or slow down the occurrence and progression of atrophic gastritis, thus reducing the risk of gastric cancer	High	Strong	100%
		S37. The eradication of *H. pylori* in patients with gastric mucosa atrophy and intestinal metaplasia can reduce the risk of gastric cancer to varying degrees, but regular follow-up should be performed	High	Strong	100%
		S38. *H. pylori* eradication therapy after endoscopic treatment of early gastric cancer or high-grade dysplasia can effectively prevent metachronous gastric cancer	High	Strong	100%
		S39. Some Chinese patent medicines can be used in the treatment of *H. pylori*	Low	Weak	92.7%
	Folic acid, antioxidant vitamins	S40. Folic acid, antioxidant vitamins, etc. may delay the process of atrophic gastritis in some people, thus reducing the risk of gastric cancer	High	Strong	100%
		S41.The combination of antioxidant vitamins and *H. pylori* eradication therapy can delay or even block the occurrence and progression of gastric precancerous lesions, thereby reducing the risk of cancer	Moderate	Strong	96.4%
	Treatment by integrated traditional Chinese and Western medicine	S42.Traditional Chinese medicine has certain efficacy in treating gastric precancerous lesions, and integrated traditional and western medicine has advantages	Moderate	Strong	94.55%
Efficacy evaluation	Research design	S43. Strict research designs, procedure quality control, and standardized report are important prerequisites for improving the level of evidence in intervention studies for gastric precancerous lesions	High	Strong	98.2%
		S44.The clinical intervention research process of precancerous lesions of gastric cancer should generally not be less than 6 months, followed by no less than 6 months of follow-up	Low	Strong	96.4%
	Positioning and goals of medical interventions	S45. The intervention of chronic atrophic gastritis should be aimed at gastric body or total gastric atrophy and/or intestinal metaplasia to promote the regression of the disease and reduce the risk of gastric cancer. Medical interventions for gastric precancerous lesions should target uncertain dysplasia and low-grade dysplasia, with the goal of promoting the reversal of the disease	Moderate	Strong	96.4%
	Key technologies	S46.The efficacy of the evaluation of dysplasia needs to be accurate and to be focused. Targeted monitoring based on MTB technology can help to improve the consistency of biopsy sites before and after treatment	Low	Strong	96.4%
	Efficacy evaluation methods	S47. The efficacy of the evaluation of gastric precancerous lesions should be based on histopathology, supplemented by a comprehensive evaluation of gastroscopy, symptoms, and quality of life	Low	Strong	98.2%
	Histological semiquantitative evaluation of dysplasia	S48. The histological semiquantitative evaluation of gastric mucosal dysplasia can be carried out from the microscopic level of cell structure atypia and gland disorders to refine the efficacy evaluation research	Low	Weak	87.3%

## Definition and epidemiology

### Definition

The development of gastric cancer is a multifactor and multistep process. The generally accepted Correa evolution model of intestinal gastric cancer is that gastric mucosa exhibits inflammation and atrophy, intestinal metaplasia (IM), and dysplasia (intraepithelial neoplasia) in steps under the influence of *H. pylori* infection and other factors and finally develops into gastric adenocarcinoma.

In 1972, the World Health Organization (WHO) classified precursors of gastric cancer into precancerous diseases and precancerous lesions and proposed the concept of precancerous lesions [11]. Gastric precancerous diseases include gastric ulcers, chronic atrophic gastritis (CAG), gastric polyps, remnant stomach, Ménétrier's disease and so on.

Gastric precancerous lesions refer to pathological changes that are easily transformed into cancerous tissues, and dysplasia is a direct precancerous lesion. Some Western scholars also classify atrophy, IM and dysplasia as broad precancerous lesions.

In 1978, the WHO approved the unified use of the term dysplasia [12] and defined its degree, which was divided into mild, moderate and severe grades.

In 2000, the WHO International Agency for Research on Cancer [13] recommended the use of the term gastric epithelial neoplasia (GIN).

In 2010, the WHO recommended that the terms dysplasia and intraepithelial neoplasia should be used equally [14]. Dysplasia focuses on morphological changes, while intraepithelial neoplasia emphasizes the process of tumor evolution, which can be divided into low-grade intraepithelial neoplasia (LGIN) and high-grade intraepithelial neoplasia (HGIN).

The updated 2019 WHO classification [15] suggested that the term gastrointestinal dysplasia be adopted, which can be divided into low-grade dysplasia (LGD) and high-grade dysplasia (HGD).

This guide is focused on gastric mucosal dysplasia and its background lesions, namely, CAG and IM.

### Epidemiology

#### Statement 1. The prevalence rate of chronic atrophic gastritis in China is relatively high and increases with age

The prevalence of CAG varies greatly in different countries and regions and is related to the *H. pylori* infection rate, environmental factors, genetic background, diagnostic methods, etc. The prevalence of CAG is positively correlated with the incidence of gastric cancer.

The prevalence of CAG in China is relatively high. In 2014, a cross-sectional survey conducted by the Society of Digestive Endoscopy of the Chinese Medical Association included 8892 patients with chronic gastritis confirmed by gastroscopy in 10 cities and 33 centers. The results showed that the pathological diagnosis rate of CAG was 25.8%, the endoscopic diagnosis rate was 17.7%, the prevalence rate of IM was 23.6%, and the prevalence rate of dysplasia was 7.3% [16]. In 2016, among the 183,426 patients undergoing gastroscopy at the Peking University Third Hospital from 1989 to 2014, CAG was detected in 22.4% of the patients, the ratio of male to female patients was 1:1.4, and the average age was 59.2 ± 14.1 years [17].

A European epidemiological study [18] found that the prevalence of CAG increased with age. In a cohort study of 389 people in South Korea, the prevalence rates of gastric antrum atrophy and gastric body atrophy were 42.5% and 20.1%, respectively. The prevalence rate of CAG increased significantly with age for both men and women [19].

Some scholars have analyzed the prevalence data from studies of CAG in unselected populations in different countries published before November 2005. Among them, 15 studies confirmed CAG by gastroscopy, and 26 studies confirmed CAG by serum pepsinogen levels. CAG is more common among elderly individuals in different regions of the world. The older population is, the higher the prevalence rate, but there is no obvious sex difference. The incidence rate of CAG in some Asian countries, including China and Japan, is higher than that in other regions of the world [20].

#### Statement 2. The occurrence of chronic atrophic gastritis is closely related to *H. pylori* infection

The occurrence of CAG is closely related to *H. pylori* infection, and the risk of CAG after *H. pylori* infection can increase by 4 times [21, 22]. Even in a population with a low- *H. pylori* prevalence (< 10%), the occurrence of IM and dysplasia is closely related to *H. pylori* infection. In a population with a high prevalence of *H. pylori* infection (> 60%), more than 80% of the *H. pylori* -positive patients had active gastritis or CAG [23, 24]. As reported by Peking University Third Hospital, the *H. pylori* infection rate in CAG patients was 26.7% [17].

#### Statement 3. The sensitivity of white light endoscopy in diagnosing atrophic gastritis is poor, and the coincidence rate with the pathological diagnosis is low

In 2014, a survey conducted by the Society of Digestive Endoscopy of the Chinese Medical Association found that, with pathological diagnosis as the "gold standard", the sensitivity and specificity of the endoscopic diagnosis of atrophy were 42% and 91%, respectively, and the coincidence rate between endoscopic and pathological diagnoses needs to be improved [16]. In a 2006 multicenter study of an asymptomatic population in South Korea (n = 25,536), the prevalence of CAG diagnosed by endoscopy was 27.1%, which was lower than that diagnosed by histology [19].

### Syndrome distribution of gastric precancerous lesions

#### Statement 4. There is a lack of consolidated standards of gastric precancerous lesions for syndrome differentiation

Most PLGC patients have no specific clinical symptoms or signs, and TCM syndrome differentiation is mainly based on the underlying disease of CAG. Deficiency carried by pathogen factors-and deficiency resulting from excess are important patterns of pathogenesis transformation [25–28] and consolidated standards of syndrome differentiation are still lacking. A large multicenter cross-sectional study (n = 1000) [28] suggested that the mixed syndrome of deficiency and excess runs through the whole process of PLGC, while IM occurs in the critical stage of deficiency, converting to an excess syndrome. Another study by the same team (n = 592) [29] further concluded that the syndrome evolving characteristic of sthenia transforming into asthenia, gradually appeared as yin deficiency and blood stasis during the process of CNAG evolving into CAG, IM, and dysplasia. A cross-sectional study (n = 1056) [30] pointed out that the syndrome of spleen and stomach weakness has the maximum relevance with gastric atrophy and IM, while the syndrome of stomach meridian blood stasis has the greatest correlation with dysplasia. Another cross-sectional study (n = 307) [31] also indicated that the syndrome of stomach yin deficiency and stomach meridian blood stasis gradually increased with the progression of gastric atrophy and IM (*P* < 0.01). Therefore, the development of PLGC is a complex gradual process from the qi to blood, eventually entering the collaterals. Blood stasis and deficiency may be the key syndrome elements in the conversion of deficiency and excess syndromes. However, the relevant conclusions need to be further confirmed by a wide-range, large-sample epidemiological investigation.

## Diagnosis and grading

### Endoscopy diagnosis

Adequately enough and high-quality preoperative preparation benefits the improvement of the detection of PLGC.

#### Statement 5. The application of defoaming agents and mucolytic agents can improve the visibility of gastric mucosa, which is helpful for detecting gastric mucosal lesions

A clear visual field of the endoscope is a prerequisite for detecting lesions and performing an accurate biopsy. Excessive gastric mucus and foam can prolong an examination time and cause missed diagnosis and misdiagnosis. The elimination of foam and the application of mucolytic agents are essential to improve the quality of an observation [32]. The improved visibility of the gastric mucosa is beneficial for the detection of minimal gastric mucosal lesions, including precancerous lesions [33].

A randomized clinical trial (RCT) showed that the amount of foam in the stomach was significantly reduced after the administration of simethicone [34].

Pronase is widely used in the removal of mucus in the upper digestive tract. The application of pronase during endoscopic washing of the gastric mucosa can reduce the thickness of mucus, which is conducive to biopsy and diagnosis [35, 36]. The combination of pronase and simethicone significantly improved the gastric mucosal visual field score (73% vs. 49%) and reduced the amount of flushing water without increasing the operation time [37]. Domestic studies have shown that simethicone combined with chymotrypsin can increase the detection rate of precancerous lesions and early gastric cancer (36.4% in the test group; 26.8% in the control group, P = 0.000) [38]. Another study (n = 720) showed that the combination of the two reagents could improve the visibility of the gastric mucosa, but there was no difference in the detection rate of lesions between the groups [39].

#### Statement 6. When gastric peristalsis severely affects the observation, proper antispasmodic treatment can improve the visual field of observation and facilitate the detection of lesions

For patients with violent gastric peristalsis, which makes it difficult to observe lesions, antispasmodics should be considered [32]. Common antispasmodic methods are as follows: (a) an intramuscular or intravenous injection of 10–20 mg of scopolamine butyrate and an intravenous injection of 1 mg of glucagon; (b) a local spray of 20 ml of 0.8% peppermint oil solution [40]. RCTs have shown that spraying peppermint oil locally has a better antispasmodic effect than intramuscular injections of scopolamine butybromide and has fewer side effects [41].

At present, there is no research that provides evidence that antispasmodic treatment improves the detection rate of precancerous lesions, and drugs such as scopolamine and glucagon have side effects, so physicians should be cautious in the clinical application of these drugs. However, patients for whom observation is severely affected by gastric peristalsis, proper antispasmodic treatment can ensure a better field of view [33].

#### Statement 7. Intraoperative sedatives are recommended for patients with severe anxiety

The purpose of sedation in digestive endoscopy for diagnosis and treatment is to eliminate the anxiety and discomfort of patients, enhance their tolerance and satisfaction, reduce the risk of injury and accidents during the operation, and create the best operating conditions for endoscopists [42, 43]. Patients who are worried or fearful about endoscopy, patients who are highly sensitive and unable to control themselves [44], and patients who undergo long-term and complicated diagnosis and treatment procedures can be sedated by an anesthesiologist after eliminating contraindications. It should be noted that sedation itself has a high risk. Therefore, medical institutions that perform sedative gastrointestinal endoscopy should meet the requirements for relevant places and equipments, including the area of the diagnosis and treatment unit, complete diagnosis and treatment equipment and monitoring/rescue personnel, drugs, etc. [45].

#### Statement 8. Conventional white-light endoscopy could be used for PLGC screening. High-definition conventional chromoendoscopy, virtual chromoendoscopy and magnifying endoscopy should be used for the diagnosis of patients who are at high risk for gastric carcinoma

Conventional white-light endoscopy is the basic method for detecting PLGC; however, the correlation between histological and conventional white-light endoscopic findings for the diagnosis of gastric precancerous conditions is poor. High-definition conventional chromoendoscopy, virtual chromoendoscopy and magnifying endoscopy should be used for the diagnosis of PLGC to improve the accuracy of disease detection. A cross-sectional study showed that high-definition white-light endoscopy (HD-WLE) had a global accuracy of 88% for the diagnosis of IM, with a sensitivity of 75% and specificity of 94% [46]. A real-time multicenter prospective study showed that the global accuracy of HD-WLE for the diagnosis of IM was 83%, with a specificity of 98% but with only 53% sensitivity [47].

Conventional chromoendoscopy with the application of dyes (indigo carmine, methylene blue, acetic acid, or hematoxylin) can improve the sensitivity and accuracy for the detection of PLGC. A meta-analysis showed a sensitivity and specificity of dye-chromoendoscopy in the detection of PLGC (atrophy, IM and dysplasia) of 90% and 82%, respectively, with these results being significantly better than those of white-light endoscopy alone (P = 0.001) [48]. Virtual chromoendoscopy, which is available at the touch of a button, can avoid the misdiagnosis of lesions caused by uneven dyeing. Compared with conventional chromoendoscopy, virtual chromoendoscopy can shorten endoscopic procedures. The sensitivity and specificity of narrow-band imaging (NBI) with magnification for the diagnosis of IM were 86% and 77%, and for dysplasia/early cancer, these values were 90% and 83%, respectively [49]. A multicenter prospective randomized study showed that even though specificities for IM were the same, the sensitivity for IM (92% vs 59%) were much higher for NBI than for HD-WLE [50]. In a randomized prospective crossover study, the accuracy of IM detection by NBI was significantly higher than that by WLE (P = 0.001) [51]. Linked color imaging (LCI) has high accuracy in the diagnosis of *H. pylori* infection, and blue laser imaging (BLI) has high accuracy in the diagnosis of atrophy. BLI-ME has high accuracy in the diagnosis of IM. NBI with the application of dyes (indigo carmine, methylene blue, acetic acid) can improve the frequency for the detection of PLGC [52–54].

#### Statement 9. The evaluation and diagnosis of *H. pylori* infection status should be included in endoscopic examinations

With the deepening of the understanding of *H. pylori* Japanese scholars have systematically summarized the endoscopic manifestations of *H. pylori* infection status. The evaluation and diagnosis of *H. pylori* -uninfected gastric mucosa, *H. pylori*-infected gastric mucosa or *H. pylori* -past infected gastric mucosa can be judged through endoscopic findings with reference to the Kyoto classification of gastritis [55]. The criteria for judgment are as follows: (a) *H. pylori*-uninfected gastric mucosa: regularly arranged collecting venules (RAC) should be observed in normal gastric mucosa on the lesser curvature side from the angular region to the lower gastric body. An enlarged fold, mucosal swelling, and sticky mucus should not be seen in noninfected gastric mucosa; (b) *H. pylori* -infected gastric mucosa: atrophy, IM, diffuse redness from the fundus to the stomach body, loss of RAC, enlarged fold, mucosal swelling, nodularity, xanthoma, hyperplastic polyp, and sticky mucus are observed; (c) *H. pylori* -past infected gastric mucosa: atrophic gastric mucosa and/or other current infection features are noted, with the following exceptions: diffuse redness from the fundus to the stomach body, mucosal swelling, sticky mucus, and an enlarged fold. After *H. pylori* eradication, map-like redness is sometimes observed. Thus, map-like redness indicates past infection with *H. pylori*.

### Biopsy

#### Statement 10. An adequate number, depth and size of biopsy samples are essential for the accurate diagnosis and evaluation of PLGC

High-quality endoscopy with biopsies is the key to increasing the accuracy of pathological diagnoses and ensuring the repeatability of pathological results, thus playing an important role in the diagnosis and evaluation of PLGC.

The European Society of Gastrointestinal Endoscopy (ESGE) stated in 2019 that CAG and IM are often unevenly distributed throughout the stomach. For the adequate staging and grading of gastric precancerous conditions, at least four nontargeted biopsies of two topographic sites (at the lesser and greater curvature, from both the antrum and the corpus) should be taken, and additional targeting biopsies of suspicious lesions should be taken [56]. The British Society of Gastroenterology (BSG) guidelines of 2019 recommended that patients with image-enhanced features of CAG undergo biopsies for confirmation of the endoscopic diagnosis; biopsies are directed at mucosal sites within the Sydney protocol areas. Biopsy samples should be labeled either directed or random to corroborate endoscopic staging assessments. Meanwhile, it also recommended that patients with LGD undergo a second endoscopy with enhanced imaging and extensive biopsy sampling, followed by repeat endoscopy within 1 year. If patients are diagnosed with HGD, they should undergo an immediate second endoscopy with enhanced imaging and extensive biopsy sampling. Extensive biopsy should also be performed for patients with an endoscopic suspicion of CAG, IM or early gastric neoplasia [56]. The Chinese Digestive Endoscopy Association consensus of 2014 and the 2017 pathology group of the Chinese Digestive Society recommended that for patients suspected to have CAG, biopsies should be taken at the incisura, the lesser and greater curvature from the antrum (2–3 cm away from the pylorus), and the lesser and greater curvature from the corpus (8 cm away from the cardia) [57, 58]. Excluding the number of biopsy specimens, the depth and size of the biopsy sample must be sufficient to reach the lamina propria layer [57].

J. G. Lash [59] evaluated 400,738 gastric biopsy sampling sets and reported that the acquisition of at least two biopsy specimens from the antrum and corpus, essentially following the Sydney System recommendations, is a sensible strategy that guarantees the maximum diagnostic yield for the most common gastric inflammatory conditions. Meanwhile, in a prospective study from a single center, a total of 1080 biopsies from 176 patients were investigated and it was found that obtaining 4 specimens may be sufficient for the accurate diagnosis of a depressed/ulcerative or polypoid gastric lesion, regardless of its histological stage. For infiltrative lesions, at least 5 to 6 biopsies per lesion, with more representative sampling from thickened mucosal folds, may be preferable [60]. Endoscopists with a higher biopsy rate had a lower risk of missed cancer and a higher PLGC diagnosis rate [61]. If CAG and IM lesions are suspected, biopsies should be taken from the antrum and corpus. Adequate numbers, depths and sizes of biopsy samples are essential for the accurate diagnosis and evaluation of precancerous lesions of gastric cancer.

#### Statement 11. Targeting biopsy of suspicious lesions is conducive to the evaluation of curative effects and follow-up monitoring

The sensitivity of endoscopy in the diagnosis of PLGC is low, and the pathological examination of biopsy samples is often needed to confirm the diagnosis. Due to the lack of accurate localization marks in the gastric mucosa, it is very common to perform biopsies from different positions and the diagnosis of lesions may be missed in routine endoscopic examinations. The marking targeting biopsy (MTB) uses specially designed calibrated biopsy forceps for biopsies, and it can perform biopsies and identify the positions of lesions at the same time [62]. The method is safe, effective and easily used in operations [63–65]. It was reported that the effective rate of marked gastric localization was higher than 95% after 12 months; after 2 years of follow-up, there was no statistical significance in the occurrence rate of gastric wall calibration marks. This result suggested that the calibration marks can be retained in the gastric wall for more than 2 years [66]. For chronic gastritis patients with OLGA/OLGIM stage III/IV or patients with a high score of gastric cancer screening, marking targeting biopsy is recommended for suspected lesions [9].

#### Statement 12. Additional biopsies are required for visible lesions and suspected neoplastic lesions under endoscopy

High discrepancy rates existed between endoscopic forceps biopsy and resected specimen for forceps biopsy, which is limited by its superficiality and small specimen size [67], while multiple biopsies can improve the diagnostic accuracy [68]. Therefore, additional biopsies were necessary to increase the diagnostic accuracy for suspicious lesions. The number of biopsies for lesions suspected to be early gastric cancer is still not unified. According to the 2014 version of the Consensus of our country on early gastric cancer screening and endoscopic diagnosis and treatment, the number of biopsies for suspected early gastric cancer lesions depends on the size of the lesions. If the maximum diameter of the lesions is > 1 cm, the number of specimens should be ≥ 2; for lesions > 2 cm, the number of specimens should be ≥ 3; and for lesions > 3 cm, the number of specimens should be ≥ 4 [69]. Multiple biopsies were related to a high risk of biopsy-induced ulcers, bleeding, and local fibrosis after multiple biopsies, which may increase the difficulty of endoscopic treatment and raise the risk of adverse events. In addition, local fibrosis may lead to a negative lift sign during endoscopic treatment, resulting in misjudgment of the lesion depth and affecting the selection of treatment options. Therefore, the consensus of our country in 2017 proposed that, for gastric mucosa suspected to be early neoplastic lesions, one to two biopsy samples for lesions less than 2 cm and an additional biopsy should be obtained for every 1 cm increase in diameter was recommended [58]. A retrospective study in Japan found that the diagnostic accuracy of two biopsies for early gastric cancer was 92.5%, which was significantly higher than that of 83.9% for one biopsy, irrespective of tumor size; however, the diagnostic accuracy did not significantly differ between two and three or more biopsies. Therefore, this study indicated that two biopsies was the optimal number required to diagnose early gastric cancer [70]. With the application of more powerful image-enhanced endoscopic techniques, such as magnifying endoscopy with narrow-band imaging (M-NBI), it is possible to avoid unnecessary biopsies and reduce the number of biopsies [71, 72], but the optimal number of endoscopic biopsy specimens for suspicious lesions still needs to be further explored.

### Pathological diagnosis and evaluation

#### Statement 13. Standardizing the protocol of biopsy specimen processing will help improve the accuracy of pathological diagnosis [73]

The standardized protocol for biopsy specimen processing is as follows: (a) after removing the biopsy tissue, the base should be adhered to a small piece of filter paper and then immersed in fixative. The volume of the fixative should be 10 times or more that of the specimen. Generally, 4% neutral buffered formalin is used for fixation for 6 to 48 h; (b) the specimens of different parts must be separated into different bottles and labeled clearly. The bottle should be transparent, drop-resistant, and have a wide/screw-mouth; (c) when receiving the specimens, the content of the application form and the labeling information of the bottle must be checked and rechecked carefully. Whether there is tissue in the bottle, and the number of tissue blocks, must be clarified; (d) the pathologist disposing of the material needs to drop eosin dye solution on the specimen and then wrap the specimen with filter paper, or a technician adds eosin dye solution to the tissue processor to observe the structure and direction of the tissue during embedding; (e) attention should be paid to the direction of the tissue when embedding, and the tissue should be placed sideways and fixed in paraffin to ensure that the mucosal layer, muscular mucosa layer, and submucosal layer are all included when sliced; (f) the instruments, blades, tweezers, countertops, and the water surface should be cleaned timely after one block is sliced to prevent misdiagnosis caused by contamination; (g) at least 6 continuous slices should be cut from the block and collected on the same glass slide.

#### Statement 14. Subtyping incomplete intestinal metaplasia has clinical significance [74–77]

Subtyping IM is usually based on morphological changes and mucin variety. Neutral mucin, sialic acid mucin and sulfated mucin can be distinguished by AB/PAS staining (Alcian Blue, pH 2.5/Periodic Acid Schiff) and HID/AB staining (High Iron Diamine/Alcian Blue, pH 2.5). According to the different mucins secreted by different cells, IM is classified into three types: type I, type II, and type III. Type I is complete metaplasia, and type II and type III are incomplete metaplasia. Previous studies have shown that type III (incomplete type) IM is associated with an increased risk of gastric cancer.

Considering that histological subtyping is helpful in assessing the risk of gastric cancer and has potential value for estimating therapeutic results and prognosis, it is advisable to identify type III IM at a minimal cost and workload. Of course, when complete GIM is found in the specimen, it should not be ignored. We need to improve the data and evidence to clarify the significance of the histological subtyping of GIM and establish the best clinical practice for the management of gastric precancerous lesions [78, 79].

#### Statement 15. Gastric dysplasia needs to be differentiated from reactive hyperplasia

When it is difficult to determine the nature of the disease as dysplasia or reactive hyperplasia, the diagnostic accuracy can be improved by the following methods: (a) continuous deep section of the tissue block, immunohistochemical staining, histochemical staining, and interoffice consultation may be helpful; (b) cases that cannot be diagnosed can be classified as indefinite for dysplasia; (c) Experienced pathologists in gastrointestinal pathology can be consulted [80, 81].

### Mucosa syndrome differentiation under gastroscopy

#### Statement 16. Mucosal syndrome differentiation under gastroscopy is an extension of inspection in traditional Chinese medicine. It focuses on discriminating gastric mucosal lesions. It is an important reference for the overall syndrome differentiation of traditional Chinese medicine and an objective basis for guiding local treatment [10]

Mucosal syndrome differentiation under gastroscopy belongs to the category of local syndrome differentiation and is a diagnostic method used to determine the pathogenesis and syndrome by analyzing the mucosa color, morphology, folds, secretion, peristalsis, microvessels, etc. By discriminating mucosal changes and other lesions before and after *H. pylori* eradication, a reasonable local application of a medication regimen can be formulated, which is also an important supplement to the overall syndrome differentiation. Due to the lack of evidence from high-quality prospective cohort studies, we referred to the syndrome differentiation criteria under gastroscopy from the Consensus of TCM Diagnosis and Treatment Experts of Chronic Gastritis (2017) (Table 2).

**Table 2:** Syndrome differentiation criteria

Syndrome	Criteria
Syndrome of incoordination between the liver and stomach	Acute active inflammatory reaction of the gastric mucosa, accompanied by bile reflux and rapid gastric peristalsis
Syndrome of spleen and stomach dampness-heat	The gastric mucosa was congested and edematous, with obvious erosion and thick and cloudy mucus
Syndrome of spleen and stomach deficiency	The gastric mucosa becomes thinner, pale or gray in color, has thin and abundant folds or mucosal edema, submucosal blood vessels are clearly visible, and gastric peristalsis is weakened
Syndrome of stomach yin deficiency	The mucosal surface is rough and uneven, thinned and brittle, and has less secretion. The folds become thinner or disappear, showing crack-like changes, or a network of small blood vessels under the mucosa can be seen through the mucosa
Syndrome of stomach collateral blood stasis	The gastric mucosa is granular or nodular, with bleeding spots in the mucosa; the mucus is gray or brown, and the vascular network is clearly visible, with dark red vascular stria

## Monitoring

### Dysplasia

#### Statement 17. Patients with dysplasia found on random biopsy should be re-evaluated by high-definition virtual or dyeing chromoendoscopy. If no visible lesions are found in the re-evaluation, the patient should be monitored once again by high-definition virtual or dyeing chromoendoscopy, with an interval of 6–12 months

High-definition virtual or stained endoscopy can improve the detection rate of dysplasia. A study included 20 patients with dysplasia or gastric cancer. Conventional endoscopy did not find visible lesions, but high-definition virtual or stained endoscopy found visible lesions in 18 of the patients [82]. Random biopsy found that the patients with dysplasia had a significantly higher risk of cancer, and the annual incidence of gastric cancer was as high as 6% [83]. Patients with dysplasia found during random biopsy should immediately go to a high-level endoscopy center for reassessment with high-definition virtual or dyeing chromoendoscopy. If high-quality endoscopy does not find clearly visible lesions, it is recommended the gastritis be staged by biopsy and high-quality endoscopic monitoring be performed every 6 months (HGD) to 12 months (LGD).

#### Statement 18. Screening and monitoring of dysplasia under endoscopy should be given great attention

A recent meta-analysis showed that the incidence of gastric cancer in patients with dysplasia is 40.36 cases/1000 person-years [84]. An endoscopic submucosal dissection (ESD) case series study showed that 24.0% and 52.7% of LGD and HGD patients, respectively, had histological deterioration after resection [85]. A meta-analysis found that 25% of the LGD patients had an escalation in pathological staging after resection, and 6.9% of them had malignant transformation [86]. A domestic study with a 10-year follow-up showed that 51.0%-78.7% of the LGD patients had reversal, and another 0.45%-14.3% had cancer [87]. In summary, for patients with dysplasia found under endoscopy, the screening and monitoring of visible lesions should be strengthened to detect and treat early gastric cancer.

#### Statement 19. Patients with uncertain dysplasia diagnosed by non-targeted biopsy can benefit from reevaluation with endoscopic examination

The biopsies of some tumorous lesions may be negative. One study [88] found that in 26.8% of the patients with indefinite neoplasm/dysplasia (IFND) on preoperative biopsy, 5.0% of the lesions were diagnosed as adenomas after resection, and 21.8% were diagnosed as early gastric cancer. In another study, 3 gastrointestinal pathologists reassessed IFND biopsy specimens, and a total of 11/46 patients were diagnosed with dysplasia (10 cases of LGD and 1 case of HGD) [89]. A retrospective study included 129 patients with IFND who underwent OLGA staging. The median follow-up was 31 months. Twenty-five OLGA stage III/IV patients were followed up, and 6 cases of LGD or HGD were found [90]. Therefore, patients diagnosed with IFND in non-targeted biopsy can benefit from re-evaluation with endoscopic intensive examination in centers with experience in early gastric cancer diagnosis and endoscopic treatment.

#### Statement 20. In patients with high-grade dysplasia, immediate high-quality endoscopic reassessment with Chromoendoscopy (virtual or dye-based) is recommended to determine whether endoscopic or surgical treatment should be performed

Patients with HGD have a high risk of simultaneous invasive cancer or the rapid progression of lesions [91]. In a group of PLGC patients, approximately 25% of the HGD patients were diagnosed with gastric cancer during the 1-year follow-up period [83]. The latest meta-analysis showed that the highest incidence of gastric cancer in patients with HGD was 186.40/1000 person-years, and that in patients with LGD and IFND was 11.25/1000 person-years [84]. Gastroscopic and histological reassessment should be performed immediately for patients with HGD, and endoscopic or surgical treatment is recommended for endoscopic visible lesions [92].

### CAG and IM

CAG and IM are independent risk factors for gastric cancer [93–96]. A Japanese study [97] showed that the cumulative 5-year incidence rates of gastric cancer of extensive CAG and IM were 1.9 ~ 10% and 5.3 ~ 9.8%, respectively. Patients with extensive atrophy or IM (antrum and corpus) or OLGA stage III/IV or OLGIM stage III/IV had a higher risk of progression to gastric cancer.

#### Statement 21. Patients with high-risk atrophic gastritis should be followed up with high-quality endoscopy or white-light endoscopy combined with biopsy every year, especially those with a family history of gastric cancer, who need more intensive follow-up.

Extensive atrophy or IM (antrum and corpus) was associated with a higher risk of progression to gastric cancer than single-site lesions [97–99]. OLGA stage III/IV, OLGIM stage III/IV, and endoscopically classified moderate-to-severe atrophy were significantly more frequent in the paracancerous tissues of gastric cancer [100]. OLGA stage IV, histological IM, and a higher classification of endoscopic atrophy were identified as independent risk factors for gastric cancer [101]. Ten percent of gastric cancer patients present familial aggregation [102]. The risk of progression to cancer in patients with first-degree relatives with gastric cancer increased by 2–10 times [103]. The risk of progression to cancer in patients with second-degree relatives with gastric cancer was also higher but was lower than that in patients with first-degree relatives [104]. The study found that patients with IM and a family history of gastric cancer progressed faster [99].

Therefore, it is recommended that the abovementioned patients be assessed with high-quality endoscopy every year, especially those with a family history of gastric cancer, who need more intensive follow-up.

#### Statement 22. Patients with low-risk atrophic gastritis should be followed up with endoscopy every 3 years, and those with a family history of gastric cancer should be followed up every 1–2 years

A study including 27,777 patients found that the incidence of gastric cancer was associated with the extent of atrophy: C-1 0%, C-2 0.3%, C-3 0.7%, O-1 1.3%, O-2 3.70% and O-3 5.3% [105]. Patients with mild atrophy restricted to the antrum have a relatively lower risk of developing gastric cancer. Endoscopy or serological screening is recommended every 3 years for these patients, but follow-up every 1–2 years follow-up is recommended for those with a family history of gastric cancer.

### Autoimmune gastritis

#### Statement 23. Patients with autoimmune gastritis may benefit from endoscopic follow-up every 3 years

Autoimmune gastritis is often accompanied by vitamin B12-deficiency anemia, which is known as pernicious anemia. The risk of pernicious anemia-related tumors (including gastric carcinoma and neuroendocrine tumors) is significantly increased [106].

Comparing 1,138,390 pernicious anemia patients to 100,000 matched individuals, it was found that individuals with pernicious anemia were at increased risk for noncardiac gastric adenocarcinoma (OR = 2.2, 95% CI 1.9–2.5) and gastric carcinoid tumors (OR = 11.4, 95% CI 8.9–14.7) [107]. A total of 21,265 patients with pernicious anemia were followed up for an average of 7.1 years [108], and it was found that the excess risk for gastric cancer distal to the heart increased with increasing follow-up duration. A recent meta-analysis with 27 studies and a total of 22,417 patients showed that the calculated pooled gastric cancer incidence rate was 0.3% per person-year, and the overall relative risk for gastric cancer in pernicious anemia patients was 6.8 (95% CI 2.6–18.1) [109].

Some studies showed that the greatest risk of gastric cancer in patients with pernicious anemia was in the first year of follow-up [110, 111]. One study [112] performed follow-up gastroscopies 3 years after the primary screening examination of 56 patients and identified 2 patients with gastric adenocarcinoma on follow-up. Another study [113] followed up a group of 27 patients for 6 to 7 years, and none of the patients developed gastric cancer. One study randomly assigned 24 patients to a 24- or 48-month follow-up interval, and gastric cancer was not found in either group [114]. Based on the above studies, we recommend follow-up endoscopy at 3-year intervals in patients with autoimmune gastritis.

### Noninvasive screening methods

#### Statement 24. PGI, PGI/II (PGR), G-17 and *H. pylori* -IgG can be used to screen CAG patients at high-risk of gastric cancer from general population

A cross-sectional study of 14,929 patients in China found that [115] the serum marker PGI/II ratio and G-17 and *H. pylori* -IgG expression were independently associated with the risk of gastric cancer (p < 0.05). A meta-analysis [116] included 20 studies and the overall sensitivity of PG, G-17, and *H. pylori* -IgG expression in the combined diagnosis of CAG was 74.7%, and the specificity was 95.6%. Another meta-analysis [117] showed that the overall sensitivity of PG I, the PG I/II ratio, G-17, and *H. pylori* -IgG expression in the combined diagnosis of gastric atrophy was 70.2%, and the specificity was 93.9%. Their sensitivity in the combined diagnosis of antral atrophy was 51.6%, and their specificity was 84.1%. For patients with a high risk of the combined detection of PG I, the PG I/II ratio, G-17, and *H. pylori* -IgG expression, further endoscopy should be performed.

#### Statement 25. Histological and serological MG7 testing can be used to assist in the screening of groups at high-risk of gastric cancer

MG7 is a specific monoclonal antibody for gastric cancer with high specificity and sensitivity. A number of studies have found that the expression level of MG7 antigens (MG7Ag) gradually increases in superficial gastritis, CAG, IM, dysplasia and gastric cancer [118–120]. The positive ratio of MG7Ag in noncancer patients was 3.0%-5.6%, and that in gastric cancer patients was 77.5%, suggesting that MG7Ag has an early alerting effect for gastric cancer. A study involving 2710 patients found that [121] the sensitivity of MG7Ag immuno-PCR for the diagnosis of gastric cancer was 77.5%, the specificity was 95.6%, and the accuracy was 73.12%. Histological or serological MG7Ag-positive patients are high-risk groups for gastric cancer and should undergo precise endoscopic examinations.

### Risk monitoring of precancerous lesions of gastric cancer by combining disease and syndrome

#### Statement 26. In carrying out risk monitoring and management with integrated traditional Chinese and Western medicine, in addition to serology, the Kimura-Takemoto classification, OLGA/OLGIM risk assessment, and TCM syndromes can be included

Syndromes are an important reference content for TCM diagnosis and treatment of diseases. The TCM syndromes of CAG and PLGC are related to the risk of cancer. The Kimura-Takemoto classification of endoscopy assesses the extent of gastric mucosal atrophy, among which the open type (type O) is associated with a higher risk of gastric cancer [105]. A study(n = 347) [122] found that the proportion of open-type CAG patients with insufficient gastric yin syndrome (19.1%), liver stomach stagnation-heat type (17.0%), gastric-collateral stasis syndrome (16.2%), and spleen stomach weakness syndrome (11.3%) was higher. A study [123] on the correlation analysis between the serum PGI/II ratio (PGR) and TCM syndrome types in 126 CAG and PLGC patients found that strong PG positivity was common in patients with gastric collateral stasis syndrome (28.6%) and spleen stomach weakness syndrome (25.0%). Severe gastric mucosal atrophy and PLGC are the most common syndromes of gastric collateral stasis and spleen and stomach weakness, which are considered to be high-risk syndromes of clinical cancer. A study [124] used logistic regression to analyze the correlation between TCM syndrome types and gastric cancer risk in 180 CAG patients showed that the TCM syndrome types were significantly related to the increased risk of OLGA, especially gastric collateral stasis syndrome (OR = 1.0 95% CI 1.6 ~ 60.7). Gastric collateral stasis may be one of the factors that aggravates disease progression. Therefore, on the basis of serology, the Kimura-Takemoto classification of endoscopy, OLGA and OLGIM risk assessment, and TCM syndromes can be included for risk assessment and management with integrated TCM and Western medicine [125]. The current research focuses on small single-center samples, and the relevant conclusions need to be further confirmed by epidemiological investigations and studies with large-scale samples from multiple centers.

## Treatment

### Orientation of intervention

#### Statement 27. Atrophy, intestinal metaplasia and obvious active inflammation can be treated by eradicating *H. pylori* (if positive) and the short-term use of proton pump inhibitors (PPIs) or gastro-protecting agents

Active inflammation is an important factor in the progression of CAG. The causes of active gastric mucosal inflammation include *H. pylori* infection, bile reflux, drugs, diet and certain kinds of lifestyles. The purposes of treatment are to eliminate the causes, relieve the symptoms and reduce gastric mucosal inflammation [7]. The available treatments include the eradication of *H. pylori* and application of mucosa-protecting agents. Studies have confirmed that after the success of *H. pylori* eradication therapy, neutrophil infiltration in the gastric mucosa disappears, and inflammation is quickly relieved [126]. Ten years after eradication, IM and atrophy can also be improved significantly [127], which helps to prevent or delay the further development of atrophy and IM [128]. Additionally, for CAG patients with active inflammation, the degree of inflammation could be significantly reduced with the application of PPIs [129, 130] or gefarnate [131]. Therefore, we recommend that patients with atrophy, IM and active inflammation be offered *H. pylori* eradication therapy (if they test positive) and the short-term use of PPIs or mucosa-protecting agents.

#### Statement 28. Chronic atrophic gastritis of the operative link on gastric atrophy (OLGA) and operative link on gastric intestinal metaplasia (OLGIM) stages III/IV are targets of internal medicine interventions

The OLGA/OLGIM grading and staging systems proposed by the International Group of Gastrointestinal Pathologists (Atrophy club) for the evaluation of gastric mucosal atrophy and IM are based on biopsy pathology and are divided into stages 0–IV. OLGA/OLGIM stages III/IV are dependent risk factors for gastric cancer [132]. The risks of gastric cancer in OLGA and OLGIM high-risk groups increased by 19.9 times and 38.2 times, respectively [133]. In a prospective cohort study of 1755 patients with a long-term follow-up, neoplastic lesions were only found in the OLGA stages III/IV group [134]. Therefore, OLGA/OLGIM stages III/IV are targets of internal medicine interventions.

#### Statement 29. Medical intervention is required for low-grade dysplasia, and endoscopy treatment is required for high-grade dysplasia and some low-grade dysplasia with visible lesions

LGD lesions are partially reversible and have been documented to spontaneously regress in 38 to 75% of cases and persist in 19 to 50% of cases. A total of 0 to 15% of LGD lesions progressed to HGD lesions or gastric carcinoma within 10 to 48 months [135]. The risk of HGD progression to gastric carcinoma is as high as 60–85%, with a median time of 4–48 months. In the first year of the initial diagnosis [84]. The discrepancy rate of the pathological diagnosis of LGD between endoscopic forceps biopsy and endoscopic resection was as high as 28.5%, and approximately 25% of LGD diagnosed with endoscopic biopsy was histologically upgraded after endoscopic resection, including gastric HGD (16.7%) and gastric carcinoma (6.9%) [86]. The risk factors for pathological diagnosis of LGD upgraded after endoscopic resection are [136–139] as follows: (a) endoscopic findings: a lesion size ≥ 10 mm, with surface redness, nodules, central depression, surface erosion or ulcers, located in the upper 1/3 of the stomach; ME-NBI showed clear demarcation line of the lesion, abnormal microstructure of the glandular opening and/or microvessels; (b)pathological characteristics: biopsy pathology indicated villous tubular or villous tissue in the lesion, and the positive expression of MUC6; (c) serology: *H. pylori* -CagA positivity, a decreased serum PG I/II ratio and hypergastrinemia; (d) other: aged > 45 years old, a family history of gastric cancer, people in areas with a high incidence of gastric cancer, residual stomach, etc.

Therefore, it is recommended that treatment and follow-up programs be required if patients have visible LGD lesions, HGD or carcinoma by endoscopy examination. If there is no visible lesion, but there is a histological showing of dysplasia by random biopsy, it is recommended that high-definition endoscopy or chromoendoscopy be used to reassess as soon as possible. If no lesions are found, regular endoscopic surveillance should be performed. If HGD was found, re-examination by endoscopy should be performed within 6 months. If LGD was found, re-examination should be performed within 12 months [5, 140, 141].

#### Statement 30. Surveillance and interventions should be included for indefinite for neoplasm/dysplasia lesions, and pathological consultation can be conducted. A biopsy can be repeated, if necessary, to confirm the diagnosis

IFND (indefinite for neoplasm/dysplasia) lesions are classified as Category 2 lesions according to the revised Vienna classification. It is not a final diagnosis in pathology but a classification when the morphological phenotype is indefinite [5, 56, 58]. IFND lesions are usually described in pathology reports either as neoplastic or nonneoplastic (reactive), which indicates regenerative atypical epithelia or atypical glands/cells [56, 142, 143]. The diagnosis of IFND is related to the quality of the biopsy. When the initial diagnosis is FIND lesions, a pathologist should first make a deep or serial pathological section, and if necessary, add Ki-67, p53 and other immunohistochemical stains to assist in diagnosis. Meanwhile, a consultation with pathologists should be conducted to improve the quality of diagnosis [5, 56, 58]. If a diagnosis is still not confirmed, a second high-quality biopsy should be performed [88, 142–144]. High-quality biopsy can be achieved in two ways: first, the number and size of the biopsies, which are limited to large lesions, should be increased; second, the resolution and clarity of the endoscopy, such as image enhancement endoscopy, should be increased to acquire a more accurate biopsy. Clinicians should combine ordinary white light endoscopy and magnifying endoscopy to observe the characteristics of the lesion, perform an accurate biopsy, and discuss with pathologists, if necessary, to confirm the diagnosis. The subsequent diagnosis and treatment are determined according to the type of final pathological diagnosis. If the lesion is too small to repeat the biopsy, endoscopic mucosal resection can also be applied to confirm the diagnosis [88, 142, 143].

#### Statement 31. Patients with HGD or early gastric cancer can be treated with a combination of Chinese and Western medicine after endoscopic treatment

There is a certain risk of delayed bleeding of artificial ulcers after the endoscopic resection of HGD and early gastric cancer. Acid suppressive drugs should be used prophylactically after the operation. At present, PPIs are often used as the first choice to prevent bleeding and promote ulcer healing. In China, most doctors recommend the continuous use of standard-dose PPIs for 4–8 weeks. For patients with risk factors for delayed healing of artificial ulcers after gastric ESD, the dosage of PPIs can be increased, the course of treatment can be prolonged or gastric mucosal protective agents can be added as appropriate [145]. As a further intervention after endoscopic therapy, traditional Chinese medicine preparation has been proven to be effective by many studies. Kangfuxin liquid has a protective effect on gastric mucosal injuries after endoscopic surgery and can effectively reduce bleeding [146–148]. Jianweiyuyang tablets have a therapeutic effect on iatrogenic ulcers [149, 150].

*H. pylori* infection in patients with early gastric cancer should be timely eradicated after endoscopic treatment [145]. Bismuth quadruple is currently recommended as the main eradication plan [151]. There is still a certain failure rate in the eradication of *H. pylori* by Western medicine. It has been reported that traditional Chinese medicine “sensitizes” other drugs and “reverses” bacterial resistance [152, 153]. Studies have shown that standard triple and quadruple therapy combined with traditional Chinese medicine antibacterial therapy can improve the eradication rate of *H. pylori* [154, 155]. Moreover, traditional Chinese medicine can significantly improve clinical symptoms, reduce adverse drug reactions and increase patient compliance. Therefore, for patients with drug-resistant bacteria or obvious side effects, the combination of traditional Chinese and Western medicine can take full advantages of both approaches and better solve the problem of *H. pylori* infection [156].

There is still a potential risk of recurrence for patients with early gastric cancer who have achieved curative resection or are relatively close to curative resection. The local recurrence rate is 0.13–1.3%, and the incidences of synchronous cancer and metachronous cancer are 4.0%-12.9% and 2.5–5.1%, respectively. The cumulative risk rates at 5 years, 7 years and 10 years are as high as 9.5%, 13.1% and 22.7% [145], respectively, so standardized follow-up and interventions are needed. CAG, IM and LGIN are often found in these patients. Modern medicine mainly provides *H. pylori* eradication and mucosal protection treatment and lacks ideal intervention measures to improve the pathological state of the stomach. At present, there are many reports on the treatment of PLGC with traditional Chinese medicine preparations [157, 158]. It was found that the combination of traditional Chinese medicine and Western medicine can better improve gastric atrophy, IM and dysplasia.

### Eliminating the risk factors

#### Statement 32. There is no definite evidence that PPIs can induce or aggravate gastric precancerous lesions such as atrophic gastritis or intestinal metaplasia, but the long-term use of PPI preparations is not recommended in clinical practice

In 2014, a survey of 8892 patients with chronic gastritis from 33 endoscopy centers showed that the detection rates of CAG, IM, and dysplasia were 25.8%, 23.6%, and 7.3%, respectively. PPIs were the most commonly used drugs in the above population [16]. It was shown in a RCT from Europe that acid-suppressive therapy in the form of PPIs maintained for 3 years facilitated neither the development of gastric glandular atrophy of the corpus mucosa nor the occurrence of IM in GERD patients [159]. Two meta-analyses based on RCTs found that, compared with placebo or H2 receptor inhibitors, the incidence of CAG or IM in patients with long-term use of PPIs did not show significant differences [160, 161]. The use of PPIs can significantly reduce the detection rate of H. pylori [162], and another study [163] showed that acid suppression may delay the recovery of gastric mucosal atrophy after H. pylori eradication. A recent study [164] from South Korea showed a 1.4 times increased risk of gastric cancer in patients who used PPIs ≥ 30 days. Therefore, the long-term use of PPI preparations is generally not recommended for patients with chronic gastritis unless there are clear indications.

#### Statement 33. A high-salt diet is a risk factor for gastric precancerous lesions. Patients with gastric precancerous lesions should avoid high-salt and pickled foods

The conclusions on the relationship between diet and gastric cancer were not consistent, but most studies showed that a high-salt diet is a risk factor for PLGC [165–169]. People who eat salted meat, or consume a high-salt diet for a long time, have a significantly higher risk of IM [165]. A study [166] from South Korea reviewed data from 60,261 adults who underwent gastroduodenoscopy as part of a health check-up and found that a salty diet was a risk factor related to PLGC in people ≥ 40 years of age. Another study [167] found that 24 h urinary sodium excretion was significantly increased in CAG patients with IM. A significant increase in the risk of disease progression was found in PLGC patients who consumed a high-sodium diet [168, 169], and this correlation was more obvious in people with *H. pylori* infection [133]. Studies [170, 171] from the Chinese population showed that a high-salt diet is a high-risk factor for IM and dysplasia, and its correlation with distal gastric dysplasia is more significant.

#### Statement 34. A history of long-term smoking significantly increases the risk of the occurrence and progression of gastric precancerous lesions. Patients with gastric precancerous lesions should quit smoking

A number of studies [172–174] have suggested that smoking is closely related to PLGC. A case–control study of American veterans showed that smoking was an independent risk factor for IM [172]. This finding was also supported by another case–control study from the northwestern part of China [170]. A study involving 7302 Chinese patients with chronic gastritis also suggested that smoking was an independent risk factor for the occurrence of PLGC [175]. In a study [176] from South Korea in which 199,235 patients without IM were followed up, a dose-dependent increase in the risk of IM in smokers was found, and this risk was significantly reduced in those who had quit smoking. This dose-dependent correlation was also confirmed in other studies [177, 178].Smoking is not only related to the occurrence of PLGC but also to disease severity. Long-term smokers exhibited a significantly increased risk of severe CAG and IM [179–181].

#### Statement 35. Bile reflux is a risk factor for intestinal metaplasia, and interventions targeting bile reflux may be beneficial to block the occurrence and progression of gastric precancerous lesions

An increased concentration of bile acid was found in the gastric juice of patients with IM [182], and the incidence of IM and the degree of gland atrophy were also significantly increased in patients with bile reflux [183]. Multiple clinical studies have shown that bile reflux results in a significantly higher risk of IM [184–186]. A study involving 30,465 patients who underwent a gastroscopy examination showed that bile reflux is a risk factor for gastric cancer and precancerous lesions [187]. Subsequent cross-sectional and prospective studies [188] further showed that bile reflux is an independent risk factor for gastric cancer and precancerous lesions.

The risk of bile reflux was found to be increased in patients with IM [189]. A multicenter RCT [190] from China showed that bile reflux could be relieved when CAG, IM, and dysplasia were improved or reversed. These findings suggested that interventions targeting bile reflux may be helpful in reversing PLGC.

### Eradication of H. pylori

#### Statement 36. The eradication of *H. pylori* can prevent or slow down the occurrence and progression of atrophic gastritis, thus reducing the risk of gastric cancer

In 1994, the WHO pointed out that *H. pylori* is a Class I carcinogen for gastric cancer and the most important controllable risk factor for gastric cancer prevention. The eradication of H. pylori can improve gastric mucosal inflammation, delay or prevent the progression of precancerous lesions, and partially reverse atrophy, thus reducing the risk of gastric cancer [136, 191]. Many large-scale, long-term and prospective clinical studies in China and abroad have shown that [192–194] the eradication of H. pylori can significantly prevent gastric cancer, and the longer the follow-up time is, the better the prevention effect. A meta-analysis including 6 high-quality RCTs [195] showed that H. pylori eradication as a primary preventive measure for gastric cancer is more in line with health economics standards in East Asian countries such as China and Japan. An effective treatment time is before atrophy or IM occurs.

A single-center, double-blind, placebo-controlled intervention study in 2020 [196] confirmed that *H. pylori* eradication can significantly reduce the risk of gastric cancer among *H. pylori* -infected people with a family history of gastric cancer in first-degree relatives.

#### Statement 37. The eradication of *H. pylori* in patients with gastric mucosa atrophy and intestinal metaplasia can reduce the risk of gastric cancer to varying degrees, but regular follow-up should be performed

Most studies have shown that eradication of *H. pylori* has difficulty reversing IM. A meta-analysis [197] showed that eradication of *H. pylori* had no significant preventive effect on CAG patients who had IM or dysplasia. However, the Shandong Linqu study showed [88] that the incidence of gastric cancer in the *H. pylori* eradication group and placebo group during 14.7 years of follow-up was 3.0 and 4.6%, respectively [196], and further confirmed that even if the patient has entered the stage of IM or dysplasia, the eradication of *H. pylori* has a certain effect on preventing gastric cancer [198]. Studies from Sweden [193] and Hong Kong, China [194], have also shown that long-term follow-up can show that *H. pylori* eradication has the effect of preventing gastric cancer in long-term follow-up. Therefore, for patients with atrophy and IM, the eradication of *H. pylori* can reduce atrophy and inflammation, delay the further development of IM, and reduce the risk of gastric cancer to varying degrees. However, for these patients, attention should be given to follow-up after *H. pylori* eradication treatment. Predicting the risk of gastric cancer through the OLGA and OLGIM staging systems or combined pepsinogen (PG) is suitable for screening populations at high-risk of gastric cancer [199] and then targeted and active endoscopic follow-up should be conducted.

#### Statement 38. *H. pylori* eradication therapy after endoscopic treatment of early gastric cancer or high-grade dysplasia can effectively prevent metachronous gastric cancer

Endoscopic mucosal resection (EMR) and ESD are currently the first choice for the treatment of early gastric cancer or HGIN, but the annual incidence rate of metachronous gastric cancer after endoscopic treatment is approximately 3%. Some studies have found that *H. pylori* eradication after the endoscopic treatment of early gastric cancer may reduce the risk of metachronous tumors [200]. There are also studies showing that *H. pylori* eradication after the endoscopic treatment of gastric neoplasms cannot reduce the occurrence of metachronous gastric cancer [201, 202]. A large, long-term, prospective RCT [203] found that the endoscopic resection of early gastric cancer or HGD can effectively reduce the occurrence of metachronous cancer (HR = 0.5, 95% CI 0.3–0.9).

#### Statement 39. some Chinese patent medicines can be used in the treatment of *H. pylori*

At present, there are few high-quality studies on the combination of traditional Chinese and Western medicine in the treatment of *H. pylori*, and most of the results are not credible due to methodological defects. Two RCTs evaluated the effect of Jinghua Weikang capsules in the adjuvant treatment of *H. pylori*. Jinghua Weikang [204] capsules combined with PPI triple therapy and quadruple therapy for 10 days showed no significant difference between the two groups (RR= 0.9, 95% CI 0.8-1.1). Another study [205] compared Jinghua Weikang capsules combined with quadruple therapy and quadruple therapy alone for 10 days, and the curative effect was not obvious (RR=1.1, 95% CI 0.9~1.2). Therefore, Jinghua Weikang capsules combined with PPI triple therapy is similar to quadruple therapy in terms of *H. pylori* eradication rates. Jinghua Weikang capsules can be used in the clinical setting instead of bismuth agents but cannot significantly improve the *H. pylori* eradication rate if combined with quadruple therapy. Another RCT included 196 cases of *H. pylori* -related chronic superficial gastritis [206] and showed that there was no significant difference in the *H. pylori* eradication rate between Weifuchun combined with quadruple therapy and quadruple therapy alone (RR = 1.1, 95% CI 1.0-1.2).

### Folic acid, antioxidant vitamins

#### Statement 40. Folic acid, antioxidant vitamins, etc. may delay the process of atrophic gastritis in some people, thus reducing the risk of gastric cancer

A meta-analysis [207] found that the intake of fruits (RR=0.8, 95% CI 0.7–0.9) and vegetables (RR=0.9, 95% CI 0.7–1.1) was negatively correlated with the incidence of gastric cancer, and the preventive effect was more significant after ≥ 10 years of follow-up.

In the general population, the intake of certain vitamins may reduce the risk of gastric cancer (RR=0.8, 95% CI 0.7–0.8) [208]. Another large cohort study [209] showed that multivitamins did not reduce the incidence of gastric cancer. A randomized intervention study in 2019 [210] showed that 3365 patients in Linqu, Shandong Province, China, were given *H. pylori* eradication treatment, vitamin supplements (vitamin C, E and selenium, intervention for 7.3 years), an allicin intervention (7.3 years) and a placebo treatment. After follow-up for 22 years, it was found that 2 weeks of anti- *H. pylori* treatment and 7 years of vitamins significantly reduced the risk of gastric cancer, and the mortality rate of gastric cancer in the three groups was also significantly reduced.

A number of randomized, double-blind, placebo-controlled trials observed the risks of folic acid and antioxidant vitamins in preventing gastric precancerous lesions, but the results were inconsistent. A multicenter, randomized, double-blind, placebo-controlled clinical trial of 216 CAG patients in China [211], who were followed up for 6 to 7 years, showed that folic acid combined with vitamin B12 ( 20 mg/day of folic acid, 1 mg per month of an intramuscular injection of vitamin B12, reduced to 2 days per week and 1 intramuscular injection every 3 months, respectively, in the following year) could reverse gastric mucosal atrophy, partially reverse IM, and even significantly reverse dysplasia at one year. Correa et al. al [212] found that β-carotene (30 mg/day) and vitamin C (1 g/day) could increase the reversal of gastric precancerous lesions (RR = 5.1, 95% CI 1.7–15.0 and RR = 5.0, 95% CI 1.7–14.4) in the groups at high-risk of gastric cancer. Other studies [213, 214] have not found that antioxidant vitamins have a protective effect on gastric precancerous lesions. Some scholars [215] suggest that effective intervention measures should aim at nutritional supplementation (i.e., a physiological dose rather than a pharmacological dose) in groups at high-risk of gastric cancer. Of course, it is also possible that the intervention and follow-up time in these studies were too short. In addition, the dosage, course of treatment, starting age and the presence or absence of interference from other factors (such as nutritional status) are very important.

#### Statement 41. The combination of antioxidant vitamins and *H. pylori* eradication therapy can delay or even block the occurrence and progression of gastric precancerous lesions, thereby reducing the risk of cancer

In the population at high-risk of gastric cancer, antioxidant vitamins combined with *H. pylori* eradication can block the progression of PLGC. Two domestic clinical intervention trials for CAG patients showed that eradication of *H. pylori* combined with folic acid could significantly improve the degree of gastric mucosal atrophy, IM and dysplasia [216, 217]. A meta-analysis including two large-scale and long-term RCTs [197] at home and abroad shows that if gastric precancerous lesions already existed before *H. pylori* treatment, *H. pylori* eradication combined with antioxidant vitamins could significantly reduce the relative risk of gastric cancer (RR = 0.5, 95% CI: 0.3–0.9).

### Treatment by integrated traditional Chinese and Western medicine

#### Statement 42. Traditional Chinese medicine has certain efficacy in treating gastric precancerous lesions, and integrated traditional and western medicine has advantages

A meta-analysis showed that Chinese herbal compounds used in the treatment of CAG with dysplasia were superior to Western medications in improving clinical symptoms and had a certain curative effect trend in improving histopathology [218, 219]. The combination of traditional Chinese medicine and Western medicine had advantages in the treatment of gastric precancerous lesions [220–222]. A study based on gastric mucosa marking targeting biopsy technology to evaluate the efficacy showed that Moluodan [190] could improve the clinical symptoms and gastric mucosal pathological status of patients with CAG with dysplasia. This study was cited by the Chinese consensus on chronic gastritis in 2017 [7] and the European guideline for the management of epithelial precancerous conditions and lesions in the stomach in 2019 [5]. In general, there is still a lack of evidence from multicenter, large sample, placebo-controlled, long-term follow-up clinical studies.

## Efficacy evaluation

The quality of PLGC clinical evaluation studies needs to be improved. These methods will help improve the quality of clinical research to generate more high-level evidence for clinical application, such as scientific positioning, rigorous designs, standardized evaluation methods and research reports.

### Research design

#### Statement 43. Strict research designs, procedure quality control, and standardized report are important prerequisites for improving the level of evidence in intervention studies for gastric precancerous lesions

A RCT is a standard design scheme for verifying the efficacy of interventions, and the quality of its research and reports directly affects the judgment of the efficacy of an intervention [223]. In recent years, an increasing number of studies on gastric precancerous diseases and PLGC have been carried out, but the quality of research is still low, which has affected the quality of the evidence [224]. The design of RCT clinical methods should be strengthened, such as random methods, allocation plan hiding, sample size estimation, blind methods, and curative effect evaluation methods [225]. In addition, the research report should be standardized in accordance with the items required by the CONSORT statement [226].

#### Statement 44. The clinical intervention research process of precancerous lesions of gastric cancer should generally not be less than 6 months, followed by no less than 6 months of follow-up

The symptoms of CAG and PLGC are recurrent, and the lesions under gastroscopic pathology also show focal and gradual migration changes. It takes 1 to 3 months to regenerate and rebuild the gastric mucosa and restore normal functions. Therefore, the course of CAG treatment should not be less than 3 months (generally 3 to 6 months) [227]. The intervention course for PLGC should be at least 6 months, followed by no less than 6 months of follow-up. Long-term follow-up should be strengthened to observe the end-point indicators, such as the incidence of gastric cancer and the monitoring of disease recurrence.

### Positioning and goals of medical interventions

#### Statement 45. The intervention of chronic atrophic gastritis should be aimed at gastric body or total gastric atrophy and/or intestinal metaplasia to promote the regression of the disease and reduce the risk of gastric cancer. Medical interventions for gastric precancerous lesions should target uncertain dysplasia and low-grade dysplasia, with the goal of promoting the reversal of the disease

Gastric mucosal atrophy, IM and LGD are independent risk factors for gastric cancer. LGD is considered a direct PLGC. Diagnostic ESD resection is recommended for LGD with endoscopic visible lesions and a clear range, while LGD with endoscopic invisible lesions is still an important objective of medical interventions. The wider the range of gastric mucosal atrophy/IM, the higher the risk of gastric cancer [56]. Severe atrophy involving the whole stomach (whether or not it is IM) has a high risk of gastric cancer. Active monitoring and interventions are needed to reduce the risk of gastric cancer.

IFND should not be considered a harmless diagnosis. It is suggested that IFND diagnosed by nontargeted biopsy should be reassessed by gastrointestinal pathologists and reexamined by high-definition endoscopy. It is recommended to perform a second endoscopy after 6–12 months if no lesions are found; it is necessary to develop a monitoring plan based on the severity of the precancerous state and the staging of the lesion range, paying special attention to OLGA stage III/IV patients if repeat nontargeted invisible lesions were found in the biopsy, and no dysplasia was found [90]. Follow-up monitoring can be combined with medical drug interventions.

### Key technologies

#### Statement 46. The efficacy of the evaluation of dysplasia needs to be accurate and to be focused. Targeted monitoring based on MTB technology can help to improve the consistency of biopsy sites before and after treatment

Gastric mucosal atrophy and dysplasia are distributed locally, and the lesions are generally small and hidden. Under white light endoscopy, they usually lack characteristic manifestations. Even if the area is indicated for the first time, it is difficult to accurately clamp at the same part during reexamination. High-definition staining endoscopy and magnifying endoscopy can improve the contrast between the lesion and normal tissue, allow for its histological characteristics to be judged according to the morphological changes of the mucosal microvessels and mucosal glandular duct openings, accurately guide the biopsy and improve the diagnostic rate. The MAPS II guidelines proposed that biopsy assisted by high-definition staining endoscopy is the best method to detect the precancerous state or precancerous lesions of gastric cancer. Mucosal calibration biopsy helps to solve the technical problem of the accurate location of lesions in follow-up monitoring and efficacy evaluations [63].

### Efficacy evaluation methods

#### Statement 47. The efficacy of the evaluation of gastric precancerous lesions should be based on histopathology, supplemented by a comprehensive evaluation of gastroscopy, symptoms, and quality of life

The impact of PLGC on patients is multifaceted, including organic changes in gastroscopy and histopathology, as well as physical pain and discomfort, psychological anxiety and panic, and a decline in work abilities and social participation abilities, eventually leading to a decline in the quality of life. Therefore, the evaluation of the curative effect for PLGC is mainly based on histopathology, supplemented by a comprehensive evaluation of gastroscopy, symptoms, TCM syndromes, quality of life, psychological evaluations and so on [227].

### Histological semiquantitative evaluation of dysplasia

The histological evaluation of dysplasia is mainly based on qualitative evaluations and can also be evaluated by combination with histological semiquantitative methods.

#### Statement 48. The histological semiquantitative evaluation of gastric mucosal dysplasia can be carried out from the microscopic level of cell structure atypia and gland disorders to refine the efficacy evaluation research.

Some scholars have used histological semiquantitative methods to diagnose PLGC, including histological atypicality (glandular crowding, irregular glands, intraepithelial folding, deep gland expansion) and cytological atypicality (nuclear polar image, nuclear stratification, nuclear shape and pleomorphism, nuclear ratio, chromatin, nucleolus, etc.) [228]. The semiquantitative evaluation method of dysplasia histology can intuitively display the morphological differences of dysplasia between samples so that it can be more sensitive to the treatment effect and can be explored for clinical research evaluation.

## Conclusion

**Fig. 1:**
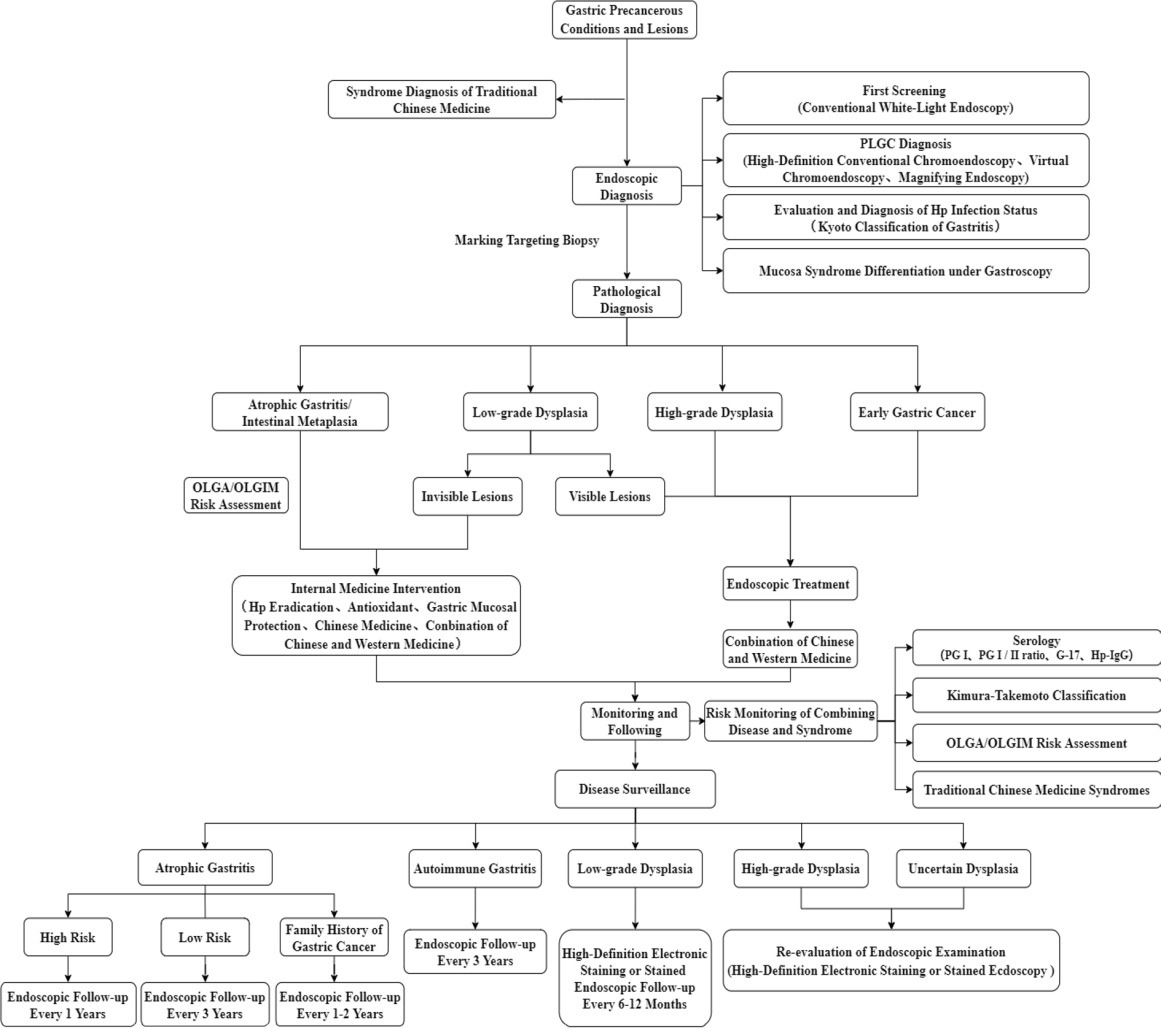
Flow chart of guide development

In China, this is the first integrated Chinese and Western medicine clinical management guide on PLGC. It has formulated detailed recommendations for the definition and epidemiology, diagnosis and staging, treatment, monitoring and follow-up of PLGC. A brief flow chart is shown in Fig. 1. Although this guide still has certain limitations, such as some recommendations lacking strong supporting clinical evidence, especially the lack of high-quality domestic research results, it still has clinical guiding significance. This guideline will play an important role in improving the standardization of the clinical diagnosis and treatment of PLGC and the quality of research.

## Data Availability

The datasets in this study are available from the corresponding author on reasonable request.

## Abbreviations

PLGC: Precancerous lesions of gastric cancer
*H. pylori*: *Helicobacter pylori*
MAPS II: Management of epithelial precancerous conditions and lesions in the stomach
TCM: Traditional Chinese Medicine
GRADE: Grading of Recommendations Assessment, Development and Evaluation
IM: Intestinal metaplasia
WHO: World Health Organization
CAG: Chronic atrophic gastritis
GIN: Gastric epithelial neoplasia
LGIN: Low-grade intraepithelial neoplasia
HGIN: High-grade intraepithelial neoplasia
LGD: Low-grade dysplasia
HGD: High-grade dysplasia
RCT: Randomized clinical trial
HD-WLE: High-definition white-light endoscopy
NBI: Narrow-band imaging
LCI: Linked color imaging
BLI: Blue laser imaging
RAC: Regularly arranged collecting venules
ESGE: European Society of Gastrointestinal Endoscopy
BSG: British Society of Gastroenterology (BSG)
MTB: Marking targeting biopsy
ESD: Endoscopic submucosal dissection
IFND: Indefinite neoplasm/dysplasia
OLGA: Operative Link on Gastritis Assessment
OLGIM: Operative link on gastric intestinal metaplasia
PG: Pepsinogen
EMR: Endoscopic mucosal resection
